# Upfront surgery vs. neoadjuvant therapy in pancreatic cancer with venous involvement: a multicenter retrospective analysis

**DOI:** 10.1007/s00423-026-04291-0

**Published:** 2026-09-22

**Authors:** Asif Halimi, Eline S. Zwart, Bengi S. Yilmaz, Benediktas Kurlinkus, Reea Ahola, Marco Del Chiaro, Ernesto Sparrelid, Elena Rangelova, Laura Maggino, Giuseppe Malleo, Gabriella Lionetto, Roberto Salvia, Keith J. Roberts, Francesco Giovinazzo, Massimo Falconi, Stefano Crippa, Giulio Belfiori, Geert Kazemier, Patrick Maisonneuve, Johanna Laukkarinen, Güralp O. Ceyhan

**Affiliations:** 1 Division of Surgery, CLINTEC, Karolinska Institutet, Stockholm, Sweden; 2 Department of Diagnostics and Intervention, Surgery, Umeå University, Daniel Naezéns väg, Umeå, 907 37 Sweden; 3 Department of Surgery, Cancer Center Amsterdam, Amsterdam UMC, Amsterdam, Netherlands; 4 Klinikum Rechts der Isar, Technical University of Munich, Munich, Germany; 5 Clinic of Gastroenterology, Nephrourology and Surgery, Faculty of Medicine, Vilnius University, Vilnius, Lithuania; 6 Department of Gastroenterology and Alimentary Tracts Surgery, Faculty of Medicine and Health Technology, Tampere University Hospital, Tampere University, Tampere, Finland; 7 Department of Surgery, University of Colorado Denver – Anschutz Medical Campus, Aurora, CO USA; 8 Department of Surgery, Section for Upper Abdominal Surgery, Sahlgrenska University Hospital, Gothenburg, Sweden; 9 Unit of General and Pancreatic Surgery, University of Verona, Verona, Italy; 10 Department of Hepato-Pancreato-Biliary and Liver Transplant Surgery, Queen Elizabeth University Hospitals Birmingham NHS Foundation Trust, Birmingham, UK; 11 Division of Pancreatic Surgery, Vita e Salute San Raffaele University, IRCCS San Raffaele Hospital, Milan, Italy; 12 Unit of Clinical Epidemiology, IEO, European Institute of Oncology IRCCS, Milan, Italy; 13 Department of General Surgery, HPB Unit, School of Medicine, Acibadem Mehmet Ali Aydinlar University, Istanbul, Turkey

**Keywords:** Pancreatic cancer, Venous involvement, Venous resection, Neoadjuvant behandling

## Abstract

**Introduction:**

The benefits of neoadjuvant therapy (NAT) for pancreatic cancer (PC) with portal venous involvement remain debated. This study aimed to compare the short- and long-term outcomes of surgery with or without NAT in patients with PC with suspected venous involvement.

**Methods:**

A multicentre retrospective cohort study compared patients who underwent upfront surgery (US) or surgery following NAT for PC with suspicion of venous involvement between 2007 and 2017. The primary endpoints were short-term morbidity and mortality, overall survival and disease-free survival.

**Results:**

We included 361 patients who received NAT and 690 patients who underwent US at nine centres. NAT patients had greater suspicion of venous involvement (96.9% vs. 83.6%, *p*<0.001), but underwent fewer venous resections (53.7% vs. 79.3%, *p*<0.001), and had less venous infiltration (52.3% vs 65.9%, *p*<0.001), perineural invasion (75.2% vs. 90.1%, p<0.001), angioinvasion (59.9% vs. 76.7%, *p*<0.001), lymph node involvement (60.6% vs. 83.6%, p<0.001) and R1 resections (44.7% vs. 67.2%, *p*<0.001) than patients who underwent US. Among patients undergoing venous resection, major postoperative complications were similar between the groups. However, without venous resection, NAT patients had significantly fewer (Clavien-Dindo ≥IIIb) postoperative complications (10.8% vs. 24.4%, *p*=0.02), fewer reoperations (3.0% vs. 11.2%, *p*=0.006), and lower 90-day mortality (2.4% vs. 7.7%, *p*=0.03). NAT patients showed improved survival (27.3 vs. 21.2 months, *p*=0.003).

**Conclusions:**

NAT maybe beneficial for patients with PC with venous involvement, as it might allow better patient selection for surgery and is associated with fewer major postoperative complications and better overall survival.

## PC and venous involvement indicate NAT

**Mini-abstract:**

Surgery outcomes for pancreatic cancer with venous involvement were compared with or without neoadjuvant therapy (NAT). NAT recipients showed improved morbidity, mortality, and overall survival. All such patients should be considered for neoadjuvant treatment.

## Introduction

Pancreatic cancer (PC) is a deadly disease with a dismal prognosis. The majority of PC patients have a metastatic disease at diagnosis and just 10–15% will have a localized disease suiting for primary resection [1–3]. However, approximately 40% of cases will display locoregional involvement of either an artery or vein [3, 4]. Although arterial involvement is a clear indication for neoadjuvant therapy (NAT), venous involvement remains a matter of debate. In fact, massive venous involvement generally results in referral to neoadjuvant therapy while minor venous involvement does not. The major concern is if venous involvement constitutes an anatomical or a biological limitation to achieve radical surgical resection.

According to the International Study Group of Pancreatic Surgery (ISGPS), patients are classified as borderline resectable if there is > 180° degrees of contact with the superior mesenteric vein (SMV) or portal vein (PV) or < 180° of contact without contour irregularity of the vein or thrombosis of the vein but with suitable vessels proximal and distal to the site of involvement, enabling safe resection and replacement [4]. For these patients, both NAT or upfront surgery (US) are recommended in the guidelines. The European Society for Medical Oncology (ESMO) and the National Comprehensive Cancer Network (NCCN) recommend that patients with borderline resectable tumours should undergo NAT before resection, as they otherwise might have a higher probability of a non-radical resection [5, 6]. The ISGPS, however, recommends US if a R0 resection is anticipated after intraoperative evaluation of tumour extent [4]. Many studies on the efficacy of NAT for resectable, borderline resectable and locally advanced tumours have recently been published [7–9]. Currently, patients with PC with suspicion of venous involvement are frequently offered NAT although the advantages are debated and results from large cohorts regarding the risks and benefits are lacking. This study therefore aimed to compare the short- and long-term outcomes in PC patients with suspicion of venous involvement receiving either US or NAT followed by surgery.

## Methods

This multicentre retrospective cohort study was conducted in accordance with the Declaration of Helsinki. The study was approved by the ethical committees of the participating centres. Depending on the centre, informed consent was obtained prior to data collection or was waived. Patients who undergoing surgery for pancreatic ductal adenocarcinoma (PDAC) or invasive intraductal papillary mucinous neoplasm (IPMN) and with suspected venous involvement on radiological imaging or intra-operatively between 2007 and 2017 were included. Patients with arterial involvement, distant metastasis, no pathological confirmation of PDAC preoperatively in case of neoadjuvant therapy or without oncological follow-up were excluded. Information regarding baseline characteristics, pre-operative treatment regimens, operation, complications, pathological outcomes, adjuvant therapy and follow-up were recorded from the patients’ files and saved in an electronic database Castor EDC [10]. Patients were divided into those undergoing US and those receiving NAT prior to surgery.

The primary endpoints were short-term (30 and 90 day) morbidity and mortality, long-term overall survival and disease-free survival. Secondary endpoints were length of stay and the correlation between radiological suspicion and true venous involvement.

The difference in the distribution of characteristics between the US and the NAT group was assessed using Student’s t-test or non-parametric test of median in case of skewed data, for continuous variables and with Pearson’s Chi Square or Fisher’s exact test for categorical variables. The cumulative incidence of relapse and of metastasis was measured from the date of surgery to the date of any first adverse event, death or last contact with the patient. Cumulative incidence functions were estimated according to Kalbfleisch and Prentice, taking competing events into account. Gray’s test was used to determine the difference between the cumulative incidence of specific events in the two groups. Overall survival was defined as time elapsing from the date of surgery to the date of death or last contact with the patient. Survival plots were drawn using the Kaplan-Meier method and log-rank test was used to assess the difference in survival between the two groups. Cumulative incidence of events and overall survival were presented at one, two, three, four and five years of follow-up. Univariable and multivariable Cox regression models were used to identify factors associated with overall survival. Statistical analysis was performed with SAS software version 9.4 (Cary, NC). All tests were two-sided and p values ≤ 0.05 were considered statistically significant.

## Results

### Patients’ characteristics

In this multicentre retrospective cohort study, 690 patients underwent US and 361 patients received NAT followed by a pancreatic resection. Patients undergoing US were significantly older (66.5 vs. 63.0 years, *p* < 0.001) and were less often smokers (22.4% vs. 33.1%, *p* = 0.002). In the preoperative diagnostics, the US patients had higher bilirubin levels (15.4 µmol/l vs. 11.5 µmol/l, *p* = 0.02), had less suspicion of venous infiltration (83.6% vs. 96.9%, *p* < 0.001) and lower degrees of contact. During the operation, patients in the US group underwent more often pancreaticoduodenectomy (76.5% vs. 68.1%, *p* = 0.01) and less often total pancreatectomy (15.8% vs. 21.3%, *p* < 0.001) than patients in the NAT group. By contrast, venous resection was more often performed in the US group (79.3% vs. 53.7%, *p* < 0.001). Wedge resection was most often performed in both groups, followed by end-to-end anastomosis (Table 1).

**Table 1:** Baseline characteristics

	Upfront surgery (US)	Neoadjuvant therapy (NAT)	*P* value*
Total, *n*	690	361	
Age, mean ± SD	66.5 ± 9.8	63.0 ± 9.4	< 0.001
Sex, Females (%)	348 (50.4)	179 (49.6)	0.79
BMI, mean ± SD	24.2 ± 3.9	24.2 ± 4.1	0.82
Currently smoking, n (%)	88 (22.4)	88 (33.1)	0.002
Pretreatment hemoglobin mg/dL, mean ± SD	8.9 ± 2.2	8.9 ± 2.2	0.89
Pretreatment CA19.9 U/mL, median [IQR]	104.6 [445.0]	82 [329.0]	0.12
Pretreatment bilirubin µmol/l, median [IQR]	15.4 [58.0]	11.5 [17.9]	0.02
Pretreatment albumin g/L, mean ± SD	35.2 ± 13.6	36.4 ± 14.6	0.36
Size of the tumor on the imaging (mm), mean ± SD	29.1 ± 10.4	29.4 ± 9.9	0.63
Suspicion of venous involvement	546 (83.6)	346 (96.9)	< 0.001
Vein infiltration in pre-operative imaging, n (%)			
0–90°	154 (28.2)	64 (18.6)	
90–180°	318 (58.2)	191 (55.4)	
180–270°	74 (13.6)	90 (26.1)	< 0.001
Preoperative drainage / ERCP, n (%)	334 (51.6)	177 (50.6)	0.75
ASA score, n (%)			0.07
I	77 (11.2)	19 ( 5.3)	
II	471 (68.3)	262 (73.0)	
III	137 (19.9)	77 (21.5)	
IV	4 ( 0.6)	0 ( 0.0)	
V	1 ( 0.1)	1 ( 0.3)	
Type of resection, n (%)			0.01
Pancreaticoduodenectomy	528 (76.5)	246 (68.1)	
Total pancreatectomy	109 (15.8)	77 (21.3)	
Distal pancreatectomy	53 ( 7.7)	38 (10.5)	
Venous resection, n (%)	547 (79.3)	194 (53.7)	< 0.001
Type of venous resection, n (%)			0.60
End to end / Segmental	193 (36.0)	75 (38.7)	
Wedge	305 (56.9)	100 (51.6)	
Autologous graft	15 ( 2.8)	7 ( 3.6)	
Synthetic graft	13 ( 2.4)	8 ( 4.1)	
Patch	10 ( 1.9)	4 ( 2.1)	

Abbreviations: *BMI* Body Mass Index, *ASA *American Society of Anesthesiologists

* Chi-square test for categorical variables, Mantel-Haenszel test for trend for ordinal variables, Student t-test for comparison of mean values and non-parametric test for comparison of median values s

Following data are missing: BMI (*n* = 263), smoking (*n* = 392), hemoglobin (*n* = 273), CA19.9 (*n* = 170), bilirubin (*n* = 262), albumin (*n* = 520), suspicion of venous involvement (*n* = 41), degree of infiltration (*n* = 1), Preoperative drainage / ERCP (*n* = 54), ASA score (*n* = 2). Type of venous resection (*n* = 11)

### Pathological outcomes

In the resected specimens, mean tumour size at 34.9 mm was significantly greater in the US group than in the NAT group at 28.2 mm. Furthermore, the pathology outcomes were worse for the US group in that the tumours were more advanced (23.1% vs. 14.2%, *p* < 0.001), showed more perineural invasion (90.1% vs. 75.2%, *p* < 0.001) and angioinvasion (76.7% vs. 59.9%, *p* < 0.001), were more often not radically removed (R1 resection 67.2% vs. 44.7%, *p* < 0.001), had more lymph node involvement (83.6% vs. 60.6%, *p* < 0.001) and more venous involvement (65.9% vs. 52.3%, *p* < 0.001) (Table 2).

**Table 2:** Pathological outcomes

	Upfront surgery (US)	Neoadjuvant therapy (NAT)	*P* value
Tumor size (mm), mean ± SD	34.9 ± 16.9	28.2 ± 16.2	< 0.001
T-stage			< 0.001
T0	0 ( 0.0)	3 ( 1.4)	
T1	117 (17.9)	118 (37.1)	
T2	386 (59.0)	152 (47.8)	
T3	151 (23.1)	45 (14.2)	
Tumor differentiation			0.18
Well differentiated	42 ( 6.4)	18 ( 8.7)	
Moderately differentiated	346 (52.6)	121 (58.5)	
Poorly differentiated	266 (40.4)	67 (32.4)	
Undifferentiated	4 ( 0.6)	1 ( 0.5)	
Perineural invasion	603 (90.1)	270 (75.2)	< 0.001
Angioinvasion	494 (76.7)	215 (59.9)	< 0.001
Resection margin			< 0.001
R0	217 (31.6)	186 (51.7)	
R1	461 (67.2)	161 (44.7)	
R2	8 ( 1.2)	13 ( 3.6)	
Lymph nodes involvement	570 (83.6)	215 (60.6)	< 0.001
Venous involvement	323 (65.9)	123 (52.3)	< 0.001

Following data are missing: tumor size (*n* = 62), T-stage (*n* = 79), tumor differentiation (*n* = 186), perineural invasion (*n* = 23), angioinvasion (*n* = 48), margin (*n* = 5), lymph node involvement (*n* = 14), venous involvement (*n* = 326)

### Morbidity and mortality

As significantly more patients in the US group underwent venous resections, short-term morbidity and mortality were divided for patients undergoing venous resection (VR+) and those not requiring venous resection (VR-). In the VR group, patients undergoing US had significantly more Clavien-Dindo grade IIIb-V complications (24.4% vs. 10.8%, *p* = 0.02), required more reoperations (11.2% vs. 3.0%, *p* = 0.006), and had a higher 90-day mortality rate (7.7% vs. 2.4%, *p* = 0.03) compared to patients receiving NAT. In the VR+ group, there were no significant differences in short-term morbidity and mortality between the US and NAT patients (Table 3). Wound infections, abscess and pneumonia proved to be the most common postoperative complications for both groups (Table 4). Complications related to venous resections are rare and did not differ statistically significantly between the two groups. Segmental (end-to-end) venous resections seemed to have a higher rate of R1 resections, but this difference was not statistically significant (Tables 5 and 6).

**Table 3:** Short-term morbidity and mortality

	No venous resection			Venous resection		
	Upfront surgery (US)	Neoadjuvant therapy (NAT)	*P* value*	Upfront surgery (US)	Neoadjuvant therapy (NAT)	*P* value
Total, *n*	143	167		547	194	
Complications, n (%)	78 (54.6)	83 (49.7)	0.39	341 (62.3)	137 (71.0)	0.03
Clavien Dindo			0.02			0.50
I-IIIa	59 (75.6)	74 (89.2)		268 (80.0)	105 (77.2)	
IIIb-V	19 (24.4)	9 (10.8)		67 (20.0)	31 (22.8)	
POPF**			0.28			0.93
No/Grade A	115 (87.1)	134 (92.4)		385 (90.2)	120 (89.6)	
Grade B	12 ( 9.1)	9 (6.2)		28 ( 6.6)	10 ( 7.5)	
Grade C	5 ( 3.8)	2 (1.4)		14 ( 3.3)	4 ( 3.0)	
PPH			0.03			0.25
No/Grade A	126 (88.1)	160 (95.8)		447 (86.1)	153 (82.7)	
Grade B	7 ( 4.9)	4 ( 2.4)		48 ( 9.3)	25 (13.5)	
Grade C	10 ( 7.0)	3 ( 1.8)		24 ( 4.6)	7 ( 3.8)	
DGE			0.40			0.37
No/Grade A	135 (94.4)	161 (96.4)		467 (89.9)	164 (88.7)	
Grade B	8 ( 5.6)	6 ( 3.6)		41 ( 7.9)	19 (10.3)	
Grade C	0 ( 0.0)	0 ( 0.0)		12 ( 2.3)	2 ( 1.1)	
Reoperation	16 (11.2)	5 ( 3.0)	0.006	53 (10.2)	21 (11.4)	0.66
Readmission	11 ( 7.7)	13 ( 7.8)	0.98	79 (15.9)	22 (12.2)	0.23
30-day mortality	5 ( 0.0)	0 ( 0.0)	0.01	15 ( 2.7)	2 ( 1.0)	0.17
90-day mortality	11 ( 7.7)	4 ( 2.4)	0.03	27 ( 4.9)	6 ( 3.1)	0.28

**Total pancreatectomies are excluded from this analysis. Abbreviations: *VR* venous resection, *POPF* postoperative pancreatic fistula, *PPH* postpancreatectomy hemorrhage, *DGE* delayed gastric emptying

Data are missing for some patients: complications (*n* = 1), Clavien-Dindo (*n* = 7), POPF (*n* = 71), PPH (*n* = 37), DGE (*n* = 36), reoperation (*n* = 36), readmission (*n* = 62)

**Table 4:** Other postoperative complications according to venous resection and treatment group

	No venous resection			Venous resection		
	Upfront surgery (US)	Neoadjuvant therapy (NAT)	*P* value*	Upfront surgery (US)	Neoadjuvant therapy (NAT)	*P* value
Total, *n*	143	167		547	194	
Complications, n (%)						
Abscess	21 (14.7)	22 (13.2)	0.70	70 (12.8)	29 (15.0)	0.45
Wound infection	7 ( 4.9)	5 ( 3.0)	0.56	33 ( 6.0)	15 ( 7.7)	0.40
Pneumonia	22 (15.4)	22 (13.2)	0.58	40 ( 7.3)	20 (10.3)	0.19
Thromboembolic	0 ( 0.0)	2 ( 1.2)	0.50	17 ( 3.1)	5 ( 2.6)	0.81
Fascial dehiscence	2 ( 1.4)	1 ( 0.6)	0.60	10 ( 1.8)	3 ( 1.6)	1.00
Kidney failure	4 ( 2.8)	0 ( 0.0)	0.04	8 ( 1.5)	4 ( 2.1)	0.52
Cardiac failure	7 ( 4.9)	6 ( 3.6)	0.58	18 ( 3.3)	4 ( 2.1)	0.47
Neurological	0 ( 0.0)	0 ( 0.0)	1.00	3 ( 0.6)	0 ( 0.0)	0.57
Venous complications, n (%)						
Thrombosis	3 ( 2.1)	0 ( 0.0)	0.10	20 ( 3.7)	6 ( 3.1)	0.82
Stricture	0 ( 0.0)	0 ( 0.0)	1.00	1 ( 0.2)	2 ( 1.0)	0.17
Bleeding	2 ( 1.4)	1 ( 0.6)	0.60	8 (10.5)	1 ( 0.5)	0.46

**Table 5:** Association between type of venous resection, resection margins, and thrombosis

		Resection margin				Thrombosis		
	Total	R0	R1	R2	*p*-value	No	Yes	
Type of venous resection, *n* (%)								
Segmental / End to end	268	74 (27.7)	187 (70.0)	6 ( 2.2)		254 (94.8)	14 ( 5.2)	
Wedge	405	144 (35.6)	255 (63.0)	6 ( 1.5)		395 (97.5)	10 ( 2.5)	
Autologous graft	22	10 (45.5)	12 (54.5)	0 ( 0.0)		21 (95.5)	1 ( 4.5)	
Synthetic graft	21	7 (33.3)	13 (61.9)	1 ( 4.8)		20 (95.2)	1 ( 4.8)	
Patch	14	4 (28.6)	9 (64.3)	1 ( 7.1)	0.28	14 (100.)	0 ( 0.0)	0.38

**Table 6:** Univariate and multivariable analysis for overall survival

Exposure Variable	Reference group	Univariate analysis*		Multivariable analysis*	
		HR (95% CI	*P*-value	HR (95% CI)	*P*-value
Male gender	(Female)	0.93 (0.80–1.07)	0.29		
Age ≥ 65 years	(< 65 years)	1.10 (0.95–1.28)	0.20		
BMI					
25–30 kg/m2	(< 25 kg/m2)	0.98 (0.82–1.18)	0.84		
>30 kg/m2	(< 25 kg/m2)	1.26 (0.90–1.75)	0.17		
Hemoglobin					
7.5–11.0 mg/dL	(< 7.5 mg/dL)	1.12 (0.89–1.41)	0.32		
>11 mg/dL	(< 7.5 mg/dL)	1.06 (0.63–1.79)	0.82		
CA 19.9 >37 U/mL	(≤ 37 U/mL)	1.08 (0.90–1.29)	0.41		
Bilirubin					
20–100 µmol/l	(< 20 µmol/l)	0.88 (0.69–1.14)	0.33		
>100 µmol/l	(< 20 µmol/l)	1.04 (0.82–1.33)	0.73		
Albumin					
35–55 g/L	(< 35 g/L)	0.98 (0.77–1.26)	0.91		
>55 g/L	(< 35 g/L)	0.84 (0.48–1.45)	0.52		
Vein infiltration scan	(Absent)	1.02 (0.82–1.29)	0.84		
Degree of contact					
90–180°	(0–90°)	1.02 (0.83–1.25)	0.87		
180–270°	(0–90°)	0.98 (0.75–1.27)	0.88		
Preoperative drainage (ERCP)	(No)	1.19 (1.02–1.39)	0.03		
Neoadjuvant treatment	(No)	0.94 (0.80–1.11)	0.47		
ASA III-V	(I-II)	1.23 (1.03–1.47)	0.02	1.25 (1.05–1.49)	0.01
Type of operation					
Total pancreatectomy	(Whipple)	1.38 (1.14–1.68)	0.0009	1.27 (1.05–1.55)	0.02
Distal pancreatectomy	(Whipple)	0.80 (0.60–1.06)	0.12		
Venous resection	(No)	1.33 (1.09–1.61)	0.004		
Tumor stage					
T0	(T1)	0.52 (0.07–3.72)	0.51		
T2	(T1)	1.16 (0.96–1.42)	0.13		
T3	(T1) / (T0-T2)	1.91 (1.49–2.46)	< 0.0001	1.49 (1.22–1.81)	< 0.0001
Lymph node involvement	(LN0)	1.73 (1.44–2.09)	< 0.0001	1.58 (1.30–1.92)	< 0.0001
Tumor differentiation					
Moderately differentiated	(Well diff.)	1.54 (1.06–2.23)	0.02		
Poorly diff./undifferentiated	(Well diff.)	2.18 (1.49–3.19)	< 0.0001	1.41 (1.20–1.67)	**< **0.0001
Perineural invasion	(Absent)	1.32 (1.05–1.65)	0.02		
Angioinvasion	(Absent)	1.14 (0.94–1.39)	0.19		
Resection margin					
R1	(R0)	1.54 (1.31–1.81)	< 0.0001	1.36 (1.15–1.61)	0.0003
R2	(R0)	1.72 (1.04–2.86)	0.03		
Venous involvement	(Absent)	1.34 (1.09–1.66)	0.007		
POST-OPERATIVE VARIABLES					
Complications	(No)	1.29 (1.11–1.51)	0.001		
POPF					
Grade B	(No/Grade A)	0.90 (0.65–1.25)	0.54		
Grade C	(No/Grade A)	2.25 (1.50–3.40)	0.0001		
PPH					
Grade B	(No/Grade A)	1.07 (0.81–1.41)	0.63		
Grade C	(No/Grade A)	1.09 (0.76–1.56)	0.63		
DGE					
Grade B	(No/Grade A)	1.46 (1.12–1.89)	0.005		
Grade C	(No/Grade A)	1.47 (0.81–2.65)	0.20		
Adjuvant therapy	(No)	0.71 (0.60–0.84)	< 0.0001		

Effect is denoted for variable values in brackets. Abbreviations: *BMI* Body Mass Index, *ASA* American Society of Anesthesiologists, *POPF* postoperative pancreatic fistula, *PPH* postpancreatectomy hemorrhage, *DGE* delayed gastric emptying

* Stratified by center ACU (*n* = 26), BIR (*n* = 32), KS (*n* = 180), MIL (*n* = 162), TAH (*n* = 34), Munich (TUM (*n* = 152), VER (*n* = 404), VUH (*n* = 15), Vumc (*n* = 46)

### Overall survival

**Fig. 1:**
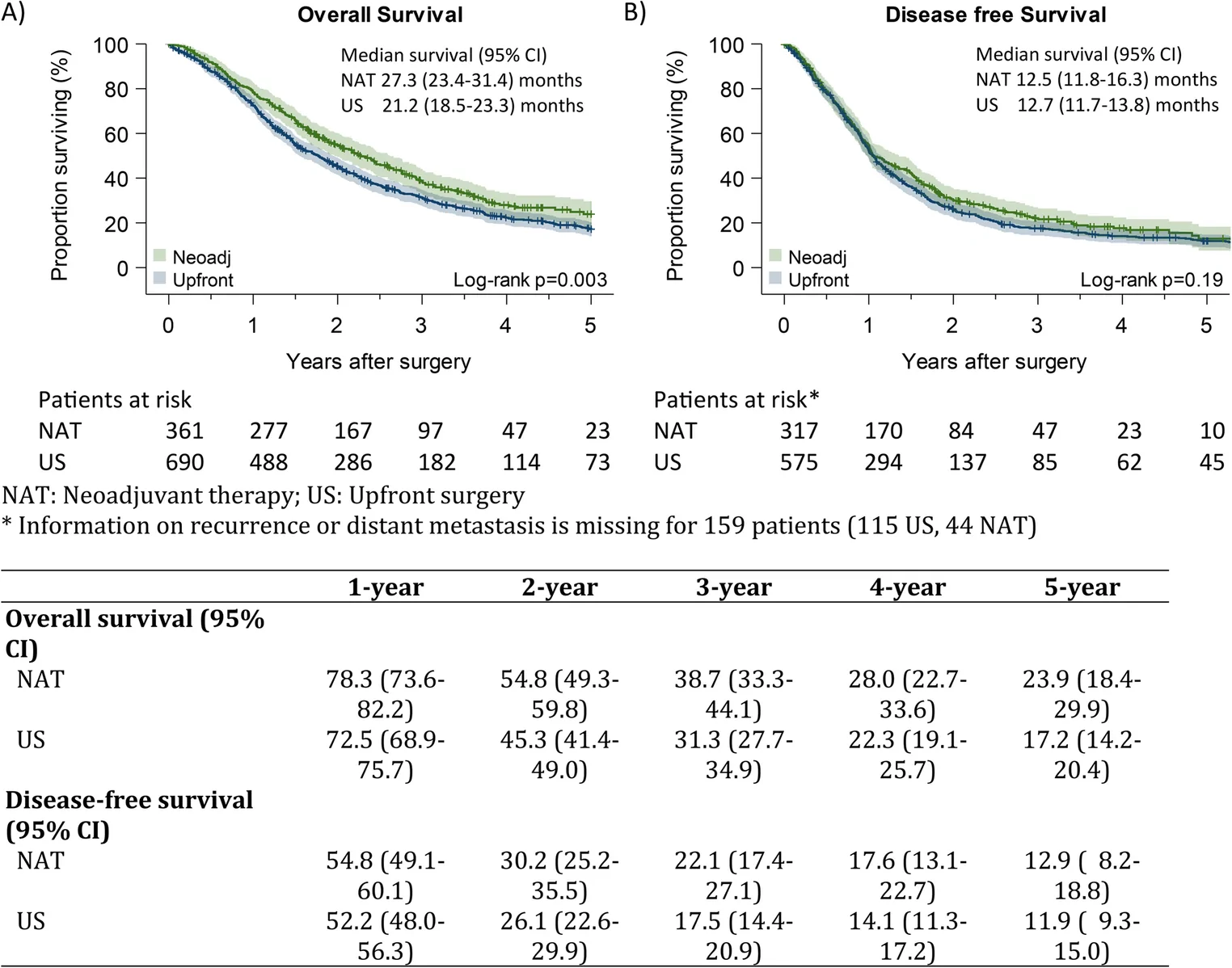
Overall survival (**A**) and disease-free survival (**B**) in upfront surgery (US) versus neoadjuvant therapy (NAT)

**Fig. 2:**
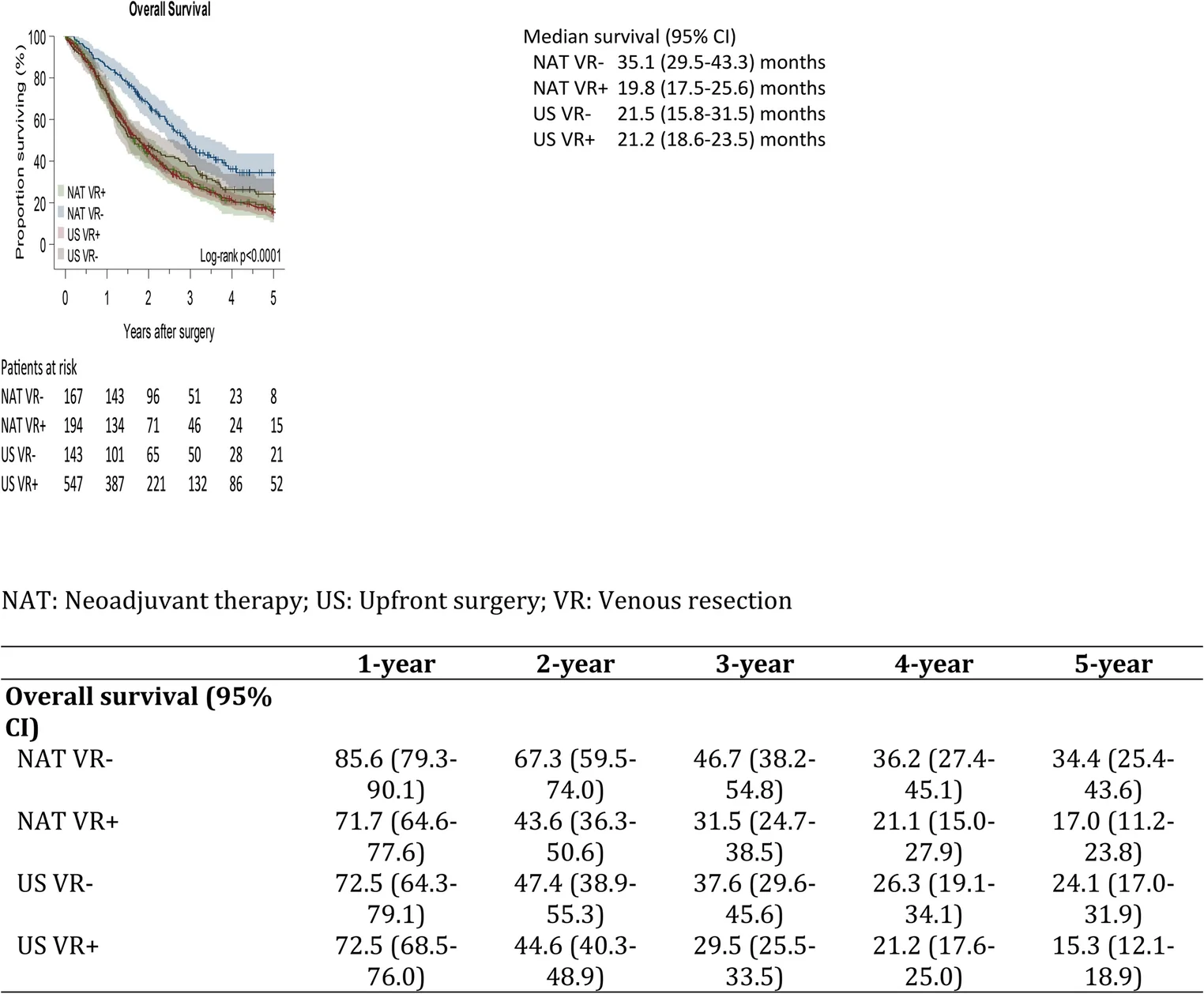
Overall survival in US versus NAT with and without venous resection

Patients receiving NAT had a significantly better overall survival of 27.3 compared to 21.2 months in patients undergoing US (*p* = 0.003) (Fig. 1a). One-year survival was 78.3% vs. 72.5%, three-year survival 38.7% vs. 31.3% and five-year survival 23.9% vs. 17.2% for patients undergoing respectively NAT and US. Significant differences remained in subanalysis taking into consideration the venous resection: patients undergoing US without venous resection had a median OS of 21.5 months while patients undergoing NAT without venous resection had a median OS of 35.1 months. Patients undergoing US with venous resection had an OS of 21.2 months, and patients undergoing NAT with venous resection had an OS of 19.8 months (*p* < 0.001) (Fig. 2).

In the univariable analysis, ASA 3–4, higher T stage, less tumour differentiation, perineural invasion, venous involvement, R1 resections, lymph node involvement, DGE and not receiving adjuvant therapy were associated with poorer survival. In the multivariable analysis, ASA 3–4, total pancreatectomy, lymph node involvement, higher tumour stage, less tumour differentiation and R1 resection were associated with poorer survival.

### Disease-free survival

In the US group, 59.2% of patients developed distant metastasis versus 63.9% in the NAT group, while disease-free survival was likewise not significantly different between US (12.7 months) and NAT (12.5 months; Fig. 1).

### Correlation between preoperative imaging, surgery and postoperative pathology

Patients undergoing US had a mean tumour size of 29.1 mm on the preoperative scan compared to 34.9 mm in their pathology reports. Patients receiving NAT had a mean tumour size of 29.4 mm preoperatively and 28.2 mm in the pathology report. Preoperative tumour size compared with size reported in the histopathology report changed more in the US group than in the NAT group (5.8 mm vs. -1.2 mm, *p* < 0.001).

Of the patients with suspected venous involvement, 65.2% underwent venous resection, and 34.8% did not. Of those patients undergoing venous resection, only 63.4% had true venous involvement according to the final pathology report. However, there were 118 patients with no suspicion of venous involvement in preoperative imaging, but they did undergo venous resection. The majority of those patients (55.1%) had true venous involvement.

## Discussion

This study showed that patients receiving NAT had better short- and long-term outcomes than US patients with fewer complications, reoperations, less short-term mortality and better overall survival.

Especially in the group with no venous resection, patients receiving NAT had less morbidity and mortality. As these patients had fewer high-grade complications, they also required fewer reoperations. Corroborating our results, it has been reported that NAT reduces the risk of major complications [11]. Furthermore, in other studies neoadjuvant chemotherapy reduced the risk of POPF, but neoadjuvant radiotherapy increased this risk [12–14]. In this study there was no significant difference in POPF [11, 15]. NAT induces fibrosis and atrophy, which leads to a firmer pancreas and thus less fistula [16]. In addition, patients receiving NAT had better short-term mortality, which was significantly different from patients without venous resection. Ninety-day mortality, which better reflects mortality after major surgery, was lower in patients receiving NAT. This may be due to less frequent POPF, but also to better patient selection as patients receiving NAT are selected patients deemed fit enough to receive combination regimens while elderly and/or patients with comorbidities may be assigned to upfront surgery, and they might only tolerate monotherapy gemcitabine.

In clinical practice, patients with larger tumours or more venous involvement will receive NAT, as in those patients it is anticipated that the tumour will be difficult to remove without NAT. By giving NAT, it is hoped that the tumour will regress, thereby facilitating surgery. The short-term outcomes in this study serve to confirm that this strategy may benefit the patients.

In addition to the short-term outcomes, median OS among patients receiving NAT was also 6.1 months longer than the US group. Only few RCTs have been published on the effect of NAT, especially when regarding venous involvement [17–19]. Reni et al. studied the effect of neoadjuvant gemcitabine, cisplatin and epirubicin. This led to an increase five-year overall survival from 13% to 24% to 49% compared to gemcitabine only or the same combination adjuvant [18]. The PREOPANC trial, also studying the effect of neoadjuvant gemcitabine and radiotherapy, also resulted in better survival after NAT, however only by 1.7 months, but this difference increased dramatically to 15.4 months when only patients undergoing resection were compared [19]. There was an especially a favourable effect on survival in patients with borderline resectable PDAC. In our study, multiple neoadjuvant treatment strategies were used. Almost half of patients received FOLFIRINOX-based therapy and a third received gemcitabine + nab-paclitaxel. These therapies were compared in the SWOG S1505 trial, where there was no significant difference in overall survival [20]. As adjuvant therapies both yielded superior results compared to gemcitabine alone [21]. The PREOPANC-2 trial found no significant survival difference between neoadjuvant FOLFIRINOX and neoadjuvant gemcitabine-based chemoradiotherapy, suggesting both regimens remain reasonable options [22].

These findings reflect an evolving and still-maturing evidence base, and should be considered when interpreting the present results, given that the treatment landscape has changed since our study period.

We could clearly demonstrate that NAT not only affects OS but also DFS. In spite of a significant difference in time to local recurrence in favour of NAT, median time to local recurrence might not be representative as most patients did not develop a local recurrence. In earlier reports, median time to local recurrence is 10.4–14.6 months [23, 24]. It is, however, important to note that fewer patients develop a local recurrence after NAT, since local recurrence is associated with shorter overall survival [25]. Many patients develop a local recurrence simultaneously with distant metastasis [26]. Furthermore, if local or distant metastasis has been detected, patients receive palliative treatment. In this phase, additional diagnostics are often not performed, hence it may be that additional distant metastasis or local recurrence are missed.

In the current study, there were 118 patients with no suspicion of venous involvement in their CT scans, but 55.1% of those patients had actual pathological venous infiltration. Therefore, it is questionable if the decision to offer NAT can be made solely on the basis of preoperative imaging. Furthermore, 18.6% had only 0–90 degrees of contact in preoperative imaging, which, according to the guidelines, would classify them as upfront resectable. However, these patients still had pathological involvement. Such patients might also benefit from NAT, as the tumours in the NAT group became significantly smaller. Reduction in tumour size may render venous resection unnecessary in some patients. In our cohort, almost all patients who received NAT had suspected venous involvement and higher degrees of contact compared to patients in the US group, but during surgery, only half required venous resection compared to 79.3% in the US group. Although lesser degree of venous involvement in radiology does not always require venous resection, NAT may have an important effect on reducing the necessity to perform a venous resection.

Several established prognostic factors, including R-status, nodal status, and receipt of adjuvant therapy, were significantly associated with survival in our cohort and were incorporated as covariates in our multivariable analysis. Although adjuvant therapy was significant in the univariable analysis, it did not remain an independent predictor after adjustment, suggesting that its apparent effect may be partly attributable to its association with other prognostic factors. Given the sample size of our cohort, further stratification of the neoadjuvant therapy effect across combinations of these variables was not feasible, as this would result in subgroups too small for reliable estimation. We therefore relied on multivariable adjustment rather than stratified subgroup analysis to account for these confounders. This should be considered when interpreting the effect of neoadjuvant therapy in the present study, and future studies with larger cohorts are warranted to further disentangle these relationships.

Survival in this study was measured from the time of surgery, which may introduce a form of immortal time bias favoring patients who received neoadjuvant therapy (NAT), as these patients must, by definition, survive the neoadjuvant treatment period and avoid disease progression in order to reach resection and be included in this analysis. This could artificially inflate the apparent survival benefit associated with NAT when compared with upfront surgery. Ideally, sensitivity analyses using time from diagnosis or treatment initiation as the time origin would help address this bias; however, complete data on diagnosis and treatment-initiation dates were not available for all patients in this retrospective, multicenter cohort. This should be considered when interpreting our survival findings, and future prospective studies incorporating these time points are warranted to further address this limitation.

A limitation of this study is that only patients undergoing surgery were included. This may have caused selection bias as patients with tumour progression during NAT did not undergo subsequent surgery were thus not included in our study. Giving NAT may cause selection of patients with favourable tumour characteristics that respond well to chemotherapy, while patients who progress might have also developed local tumour recurrence or metastasis if only adjuvant therapy was given. This cohort therefore represents a biologically and clinically preselected group, and the observed survival benefit may partly reflect this selection rather than a true treatment effect. Data on NAT dropout rates, regimen details, completion rates, and treatment response were not consistently available in this retrospective, multicenter cohort, limiting our ability to fully characterize this selection. These findings should be interpreted as reflecting outcomes among patients who completed NAT and underwent resection, and future prospective studies with complete intention-to-treat data are needed to clarify the true treatment effect of NAT.

Another possible selection bias is in the difference of patient selection. Patients receiving NAT often receive this due to suspicion of venous involvement in radiological imaging, while patients undergoing US more often have suspected venous involvement during surgery. In this study, almost all patients in the NAT group had suspicion of venous involvement in radiological imaging compared to 83.6% in the US group. Furthermore, our inclusion period was from 2007 until 2017. In those years, the outcomes of patients with pancreatic cancer improved due to centralization, better surgical techniques and better chemotherapeutic options such as FOLFIRINOX [27, 28]. Finally, as neoadjuvant treatment has been recommended in the ESMO guidelines since 2015, 32.5% of the US group were included from 2015 to 67.4% of the neoadjuvant treated group. Those patients might have better outcomes due to the period in which they underwent surgery, thus leading to bias.

To conclude, patients with suspicion of venous involvement who undergo NAT have better short- and long-term outcomes. Furthermore, preoperative imaging does not always correlate with actual pathological venous involvement. Therefore, patients with borderline resectable and also those with suspicion of any venous involvement may undergo neoadjuvant treatment. These findings are aligned with a recent and more biologically oriented trend in the treatment of pancreatic cancer than only focusing on local anatomical status [29]. 

## Data Availability

Data available upon reasonable request to the corresponding author.
